# Selma and Lois DeBakey: Icons of Medical Preservation

**DOI:** 10.14797/mdcvj.1086

**Published:** 2022-03-14

**Authors:** Jeffrey S. Reznick

**Affiliations:** 1 National Library of Medicine, National Institutes of Health, Bethesda, Maryland, US

**Keywords:** communication, preservation, history, humanities

## Abstract

**INTRODUCTION**

In his 2016 article published in this journal, Dr. William Winters described Selma and Lois DeBakey as “icons of medical communication” who believed that “nothing hinders communication as much as words, when they are used badly or incorrectly.”^1^ This article bookends Winters’ description by explaining how Selma and Lois DeBakey were also “icons of medical preservation” who asked, “Shall we nourish the biomedical archives as a viable and indispensable source of information, or shall we bury their ashes and lose a century or more of consequential scientific history?”^2^ In addressing this question posed by Selma and Lois DeBakey and spotlighting their answers in their own engaging words, we highlight the relevance of their advocacy for the medical humanities and its influence to inform humanistic approaches to science and medicine. More broadly, their advocacy inspires us to appreciate the historical record as we think critically about how we communicate the experience of medicine and science, learn from it today, and preserve it for tomorrow.

## ADvocates for Plain Language

**Figure 1:**
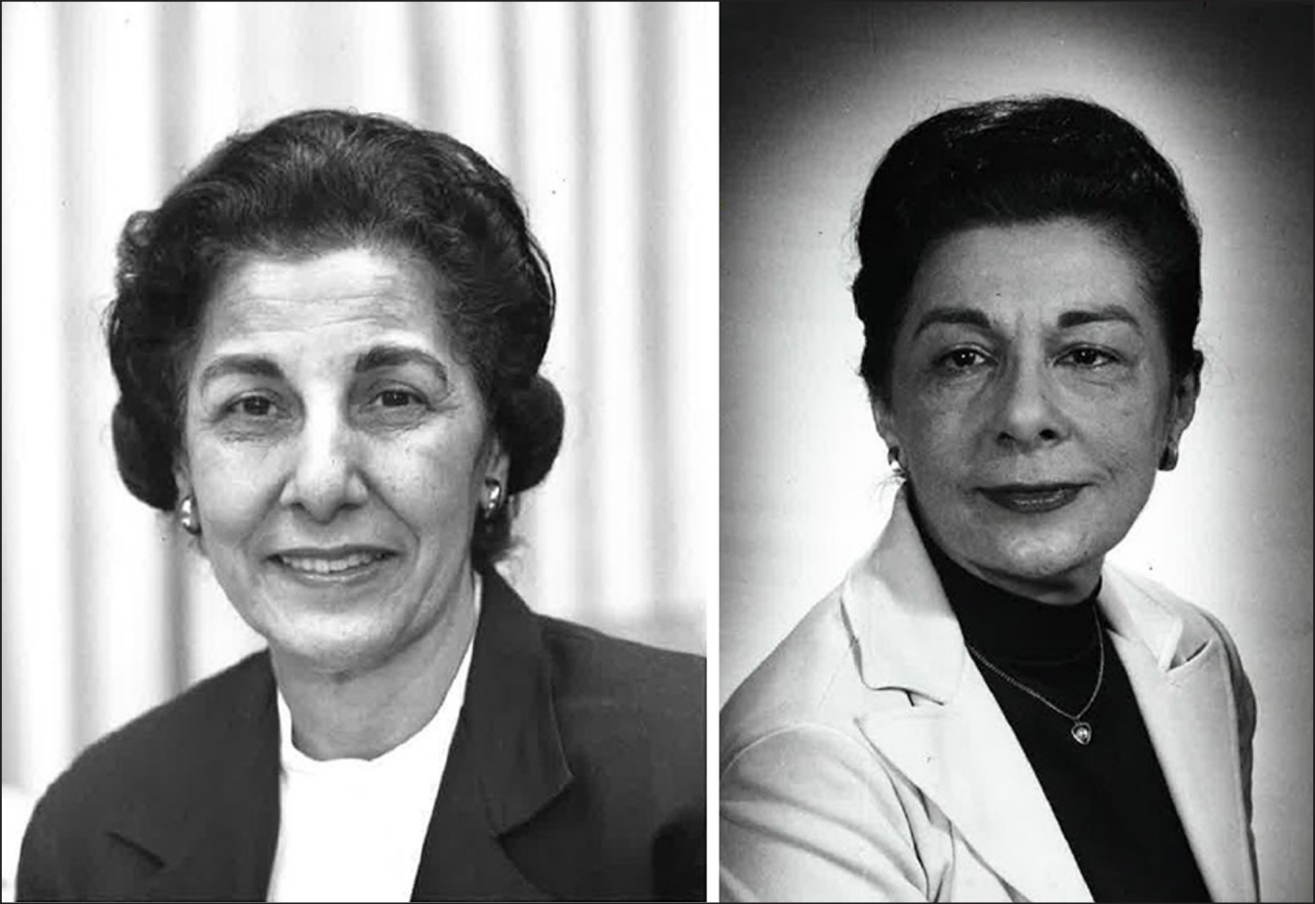
Selma and Lois DeBakey. Courtesy National Library of Medicine, National Institutes of Health.

Selma and Lois DeBakey (***Figure 1***) were not merely passionate about clear language in biomedical communications; they were ardent, unwavering, and provocative advocates. They intended their frequent hyperbolic and humorous, if not sometimes sarcastic, approaches to calling out poor communication as a means to demonstrate the absurdity of that communication and the need to remedy it for the sake of the communicators and those they were trying to inform.

Together and independently, the two sisters published many finely crafted articles in which they demonstrated their deep reading, keen interpretations, and creative ways to teach physicians and other medical professionals how to write well-organized, coherent prose free of jargon and clumsy grammar as well as the implications of not communicating plainly.^3^ Lois’ 1981 video lecture, “Doctor, are you speaking in tongues?” exemplified this distinctive body of work in a unique multimedia format that was ahead of its time.^3^

Produced by Lois for the communication courses she and Selma taught at Baylor College of Medicine and around the world, “Doctor, are you speaking in tongues?” featured Lois speaking directly into the camera about different examples of unclear biomedical texts.^4^ [***Figure 2***]

**Figure 2:** Lois DeBakey in her 1981 video lecture “Doctor, are you speaking in tongues?” Courtesy National Library of Medicine, National Institutes of Health. *https://collections.nlm.nih.gov/catalog/nlm:nlmuid-101708904-vid*

Taken together, she argued, this work:

illustrates an affliction of epidemic proportion among today’s presumably educated people. What may be called in the lingo characterizing it, lalopathy, logorrhea, verbigeration, or glossolalia is not confined to the biomedical disciplines, but extends into business, the trades, government, and all professions. In medicine, I call this disabled language “Medicant.” Ironically, the malady seems to be “pedogogenic,” induced by educational institutions. It’s highly contagious, passing freely from teacher to student, speaker to listener, and writer to reader.

**Figure 3:**
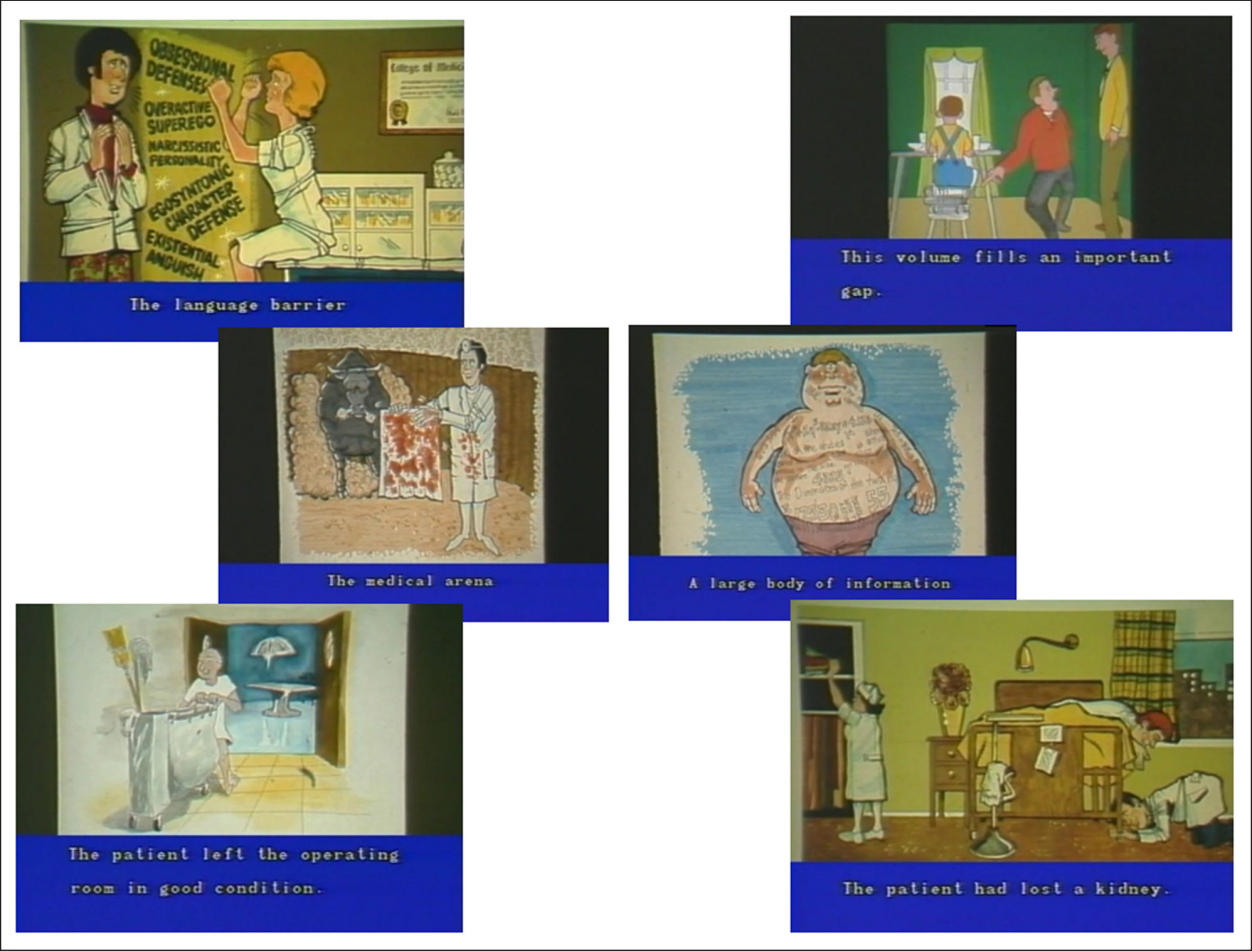
Cartoons by Dick Putney of the *Houston Post*, commissioned by Lois DeBakey, as they appear throughout her 1981 video lecture “Doctor, are you speaking in tongues?” Courtesy National Library of Medicine, National Institutes of Health. *https://collections.nlm.nih.gov/catalog/nlm:nlmuid-101708904-vid*

Later in the video, Lois utilized cartoons she commissioned from Dick Putney of the *Houston Post* to illustrate various double entendres and misused phrases that she believed too often plagued biomedical communication. Along with the video, these images became a signature teaching technique used by both sisters (***Figure 3***).

## Advocates for Preservation

While Selma and Lois were concerned about communicating in plain language, they were equally concerned with its preservation—indeed, the very physical paper upon which it was written and published. The arguments they asserted three decades ago regarding paper are equally relevant today, with ephemeral digital publications being just as vulnerable to loss as the fragile paper of the previous century. As they wrote in their seminal article, “Our Silent Enemy: Ashes in Our Libraries,”

Scholars, scientists, physicians, other health professionals, and librarians face a crucial decision today: shall we nourish the biomedical archives as a viable and indispensable source of information, or shall we bury their ashes and lose a century or more of consequential scientific history?^2^

To this question, the sisters posed clear and convincing facts:

Biomedical books and journals published since the 1850s on self-destructing acidic paper are silently and insidiously scorching on our shelves. The associated risks for scientists and physicians are serious—incomplete assessment of past knowledge; unnecessary repetition of studies that have already led to conclusive results; delay in scientific advances when important concepts, techniques, instruments, and procedures are overlooked; faulty comparative analyses; or improper assignment of priority.

They continued:

The archives also disclose the nature of biomedical research, which builds on past knowledge, advances incrementally, and is strewn with missteps, frustrations, detours, inconsistencies, enigmas, and contradictions. The public’s familiarity with the scientific process will avoid unrealistic expectations and will encourage support for research in health. But a proper historical perspective requires access to the biomedical archives. Since journals will apparently continue to be published on paper, it is folly to persist in the use of acidic paper and thus magnify for future librarians and preservationists the already Sisyphean and costly task of deacidifying their collections.

Further, they argued:

Our plea for conversion to acid-free paper is accompanied by an equally strong appeal for more rigorous criteria for journal publication. The glut of journal articles—many superficial, redundant, mediocre, or otherwise flawed and some even fraudulent—has overloaded our databases, complicated bibliographic research, and exacerbated the preservation problem. Before accepting articles, journal editors should ask: *If it is not worth preserving, is it worth publishing?*

And they concluded:

It is our responsibility to protect the integrity of our biomedical records against all threats. Authors should consider submitting manuscripts to journals that use acid-free paper, especially if they think, as most authors do, that they are writing for posterity. Librarians can refuse to purchase journals published on acidic paper, which they know will need restoration within a few decades and will thus help deplete their budgets. All of us can urge our government to devise a coordinated national conservation policy that will halt the destruction of a century of our historical record. The battle will not be easy, but the challenge beckons urgently. The choice is ours: we can answer the call, or we can deny scientists, physicians, and historians the records they need to expand human knowledge and improve health care.

Selma and Lois proceeded to argue their case methodically, beginning with the key question, “Why is preservation necessary?” and answering it convincingly with perspectives steeped in the humanities:

For intellectual, historical, social, cultural, political, and economic reasons, among others. One of the characteristics that distinguishes the human race from other members of the animal kingdom is the ability and desire to record information for posterity—to produce a collective memory that spans centuries, cultures, and national borders. Paper has been a primary medium for recording the history of our civilization, so its durability is of paramount importance. The benefits humanity derives from this historical record are numerous and well known—evident not only in more creature comforts and aesthetic and intellectual pleasures from art, literature, and music but also in our improved health and wellbeing. As Cicero aptly stated, “History is the witness that testifies to the passing of time; it illumines reality, vitalizes memory, provides guidance in daily life, and brings us tidings of antiquity (De Oratore, II, 55 BC).”

Selma and Lois also began to answer their next question, “Why preserve scientific publications?” with a distinctive humanistic perspective, stating that “the past as prologue” means

to ignore the silent enemy in our midst is to degrade the work and wisdom of our predecessors. To consign past ideas and observations to passive euthanasia as we exult over the wonders of modern high-technology is presumptuous; without past knowledge, those wonders would not have occurred. How do we place present knowledge in proper perspective if we go blindly forward, in loose-cannon fashion, without absorbing, assessing, and assimilating all previous knowledge on a subject? The proliferation of such isolated observations without proper interpretation may massage the egos of individual workers but ill serves science and humanity.

Among the specific reasons why libraries and the medical profession should preserve scientific publications was their “historical value,” Selma and Lois argued:

In the pages of the scientific archives are much of interest and value to historians: human drama—of the psychosomatically, chronically, and gravely ill; of scientific rivalry, deceit, and bitter debate; of the courageous who self-experimented or toiled day and night in tiny, ill-equipped laboratories, patiently pursuing some scientific truth….

Additionally, they argued, the preservation of scientific publications needs to happen for the sake of “bibliographic research,” because “…the responsible investigator begins each prospective research project with a thorough bibliographic search of previous publications on the subject under study.” It also needs to happen to help the biomedical scientific enterprise “avoid dangers of overlooking previous work,” and for the sake of the greater good, encompassing “editors, reviewers, the press, and the public.”

## Answering the Call

Together, Selma and Lois answered the call to preservation by advocating for the use of acid-free, permanent paper within the medical publishing industry to preserve medical records for future generations. Lois herself served as an expert consultant to the National Library of Medicine (NLM), whose congressionally mandated mission involved—and still involves—preserving biomedical communication in all its forms. At the NLM, she influenced the establishment and achievements of its Permanent Paper Task Force, which grew out of the institution’s earlier strategic planning around preservation of its collections.

**Figure 4:**
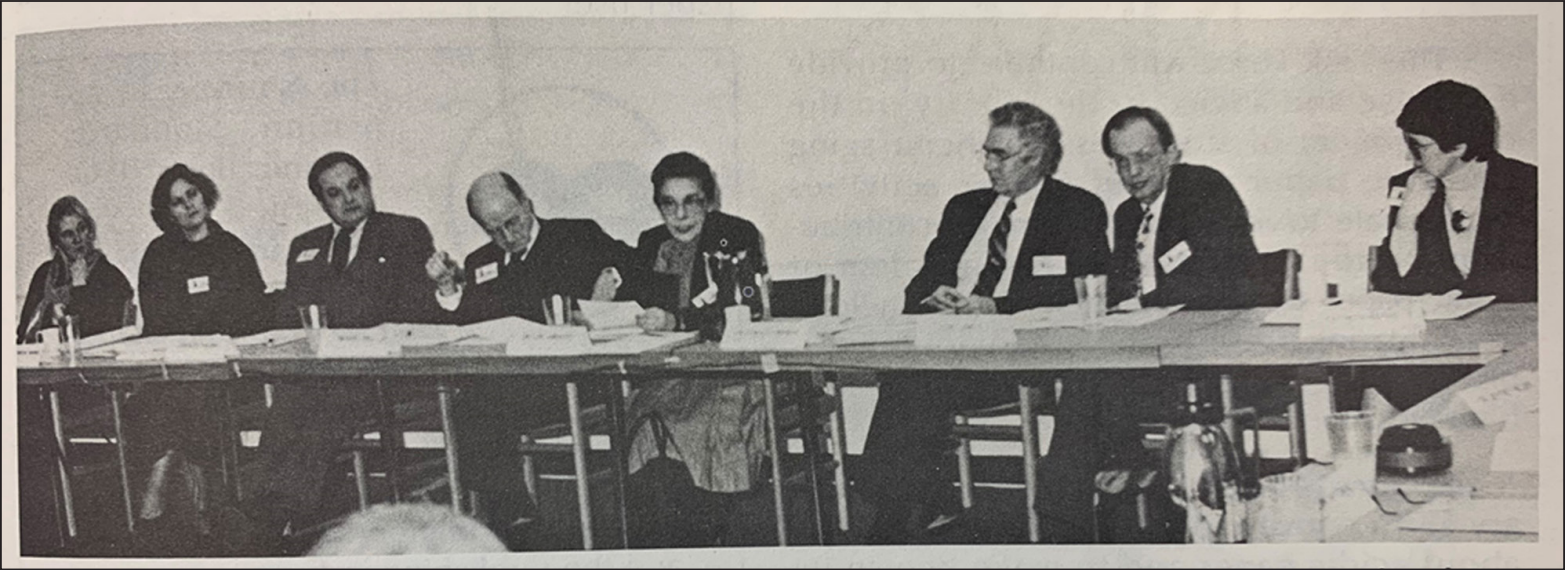
Members of the NLM Permanent Paper Task Force (from left): Heidi Kyle, conservator, Philosophical Library, American Philosophical Society; Carolyn Morrow Manns, National Preservation Program specialist, Library of Congress; Charles R. Kalina, special projects officer, NLM; Gerard Piel (chair of the Task Force), chairman emeritus, *Scientific American*; Lois DeBakey, PhD (cochair); Donald A.B. Lindberg, MD, director, NLM; Kent Smith, deputy director, NLM; Patricia R. Harris, executive director, National Information Standards Organization. NLM: National Library of Medicine. Photograph from *National Library of Medicine News* 43:2 (February 1988), p 3. Courtesy National Library of Medicine, National Institutes of Health.

Established in 1987 and cochaired by Lois and Gerald Piel, chairman emeritus of *Scientific American*, the Task Force was composed of commercial, academic, and professional society publishers as well as editors, authors, paper manufacturers and distributors, printers, librarians, and preservationists (***Figure 4***).

The group sought to make publishers and printers aware of the problems of acidic paper use, and its solutions, to help authors and editors with their concerns about making their works lasting by using acid-free paper, to alert professional societies in biomedicine and other disciplines to the need for permanence of their publications, and to encourage the application of realistic standards in the making and use of permanent paper. The Congressional *Report on Progress in Implementing National Policy on Acid-Free Paper* published in 1992 attested to the achievements of this initiative: “The efforts of the NLM Permanent Paper Task Force [having been] met with gratifying success, noting that, as of October 1991, one half of the more than 3000 of the world’s leading biomedical journals indexed by NLM are acknowledged by their publishers to be using acid-free paper, up from less than four percent in 1987.” Moreover, the report offered that “those journals are the annual equivalent of 1.1 million pages so far that will not have to be eventually microfilmed, a cost avoidance of the order of $200,000 each year at NLM,” that “four-fifths of the indexed American medical journals are now acid-free,” and that “the trend to greater acid-free paper utilization can be expected to continue as the advantages of the economics and technology of alkaline papermaking are becoming reflected in the paper market.”^5^

## Conclusion: Humanists, Both

In their advocacy for biomedical communication—access to it via plain language, and preservation of the very paper on which this language was printed—Selma and Lois were provocative, pragmatic, unrelenting, and impactful. More fundamentally, they were humanists to their respective cores, believers in the very agency of human beings and, in particular, biomedical professionals, to think critically and proceed empirically in biomedicine so its outcomes could be as meaningful and impactful as possible to society. Indeed, Selma and Lois together appreciated what humanistic thinking and action could contribute to the field of biomedical communication: to help preserve not only the “passion for medicine”—as Lois entitled her 1987 coauthored book with Phil Manning^6^—but also the knowledge and research of the field communicated in printed form that would shape the future historical record and the experience and expertise of future generations of biomedical professionals. In our expanding digital age, as paper gives way to the complex and proliferating ecosystem of born-digital material in the field of biomedicine and every other field, the advocacy of Selma and Lois DeBakey holds intellectual currency for the medical humanities and its influence to inform humanistic approaches to science and medicine. More broadly, their advocacy inspires us to appreciate the historical record as we think critically about how we communicate the experience of medicine and science, learn from it today, and preserve it for tomorrow.
